# Magnetic Resonance Imaging Assessment of Morphological Changes and Molecular Behavior to Evaluate Treatment Response of Brain Metastatic Lesions After Stereotactic Radiosurgery

**DOI:** 10.7759/cureus.73630

**Published:** 2024-11-13

**Authors:** Jorge A Garcia-Rizk, Maria F Ortiz Haro, Lourdes Noemi Santos Aragon, Dolores de la Mata-Moya, Mariana Hernandez Bojorquez

**Affiliations:** 1 Radiology, ABC Medical Center, Mexico City, MEX; 2 Radiotherapy, ABC Medical Center, Mexico City, MEX

**Keywords:** brain, magnetic resonance imaging, neoplasm metastasis, radiosurgery, treatment's response

## Abstract

Background: Brain metastases (BMs) are the most common type of intracranial tumors, frequently arising from primary cancers such as lung, breast, melanoma, and renal cell carcinoma. Magnetic resonance imaging (MRI) plays a crucial role in assessing both the morphological and molecular characteristics of BMs, particularly in evaluating treatment response following radiosurgery. However, the interpretation of these imaging changes remains complex, often influencing clinical decision-making.

Objective: This study aims to evaluate the morphological changes and molecular behavior of BMs postradiosurgery using MRI to assess treatment response.

Materials and methods: A retrospective review was conducted at a high specialty medical center, including 41 patients with BMs treated with stereotactic radiosurgery (SRS) from 2018 to 2022. Patients had a baseline MRI (pre-SRS) prior to treatment and follow-ups at 2-3 months (MRI-2) and 5-6 months (MRI-3). The response assessment in neuro-oncology brain metastases (RANO-BM) criteria were used, and T1/T2 matching was analyzed for each follow-up. Logistic regression was performed relating the T1/T2 matching and susceptibility areas (susceptibility-weighted imaging (SWI)) for MRI-2 and MRI-3. Cross tables were created regarding treatment response and demographic characteristics according to Pearson's Chi-squared test.

Results: The mean age was 56.7 years; 53.7% (n = 22) were female. Primary tumors included lung (29.3%, n = 12), breast (19.5%, n = 8), colon (12.2%, n = 5), and melanoma and kidney tumors (7.3%, n = 3). Post-SRS changes included transitions from solid to cystic lesions, reduced perilesional edema, size reduction, and increased areas of magnetic susceptibility. A mixed pattern (areas of T1/T2 match + mismatch) was noted at lesion margins during follow-ups (MRI-2: 70.7% (n* *= 29), MRI-3: 68.3% (n* *= 28)). Most patients exhibited a partial response at MRI-2 (43.9%, n = 18), while at MRI-3, disease progression occurred (43.9%, n* *= 18) due to an increase in lesion number. Logistic regression linking T1/T2 matching and SWI demonstrated a significantly central-peripheral SWI distribution for T1/T2 match during both follow-ups (MRI-2: p = 0.005, R2: 0.52; MRI-3: p = 0.002, R2: 0.56). SWI distribution was higher when a mixed T1/T2 matching was present. Significant associations were found with systemic treatment and response type at MRI-2 (p =0.001), predominantly showing a partial response for those receiving chemotherapy + targeted therapy.

Conclusions: SWI and T1/T2 mismatch are valuable tools reflecting changes in the tumor microenvironment postradiosurgery, aiding in treatment response monitoring. The appearance of susceptibility areas may precede changes in the enhancement of the lesion margin. Short-term follow-ups (2-3 months) are crucial due to prevalent progression, marked primarily by the appearance of new lesions in approximately 50% of patients.

## Introduction

Brain metastases (BMs) are the most common intracranial tumors, with an approximate incidence of 9%-30% [1,2] and a prevalence of 8.5%-10% [3,4]. The most frequent primary cancers are lung, breast, melanoma, and renal cancers [5]. They have a poor survival rate of 8.1% at two years and 2.5% at five years postdiagnosis [2].

Magnetic resonance imaging (MRI) is used for screening, diagnosis, and follow-up of patients with BMs [2]. MRI detection protocols such as those from the response assessment in neuro-oncology brain metastases (RANO-BM) and brain tumor imaging protocol (BTIP) groups recommend T1-weighted 3D pre- and postcontrast sequences, high-resolution T2, and fluid-attenuated inversion recovery (FLAIR) sequences for the assessment of vasogenic edema. To detect lesions < 5 mm, a 3 Tesla magnetic field is recommended [6].

The susceptibility-weighted imaging (SWI) sequence detects melanin and blood products due to their paramagnetic effect. SWI is sensitive to microvascular changes related to endothelial cell death postradiosurgery [7-9]. Diffusion-weighted imaging (DWI) is a technique that evaluates molecular function and microarchitecture by measuring the diffusion of water molecules. In extracellular environments, molecules diffuse freely, while in intracellular locations, they experience restriction. This distribution is affected by pathologies such as malignant neoplasms, infarcts, and abscesses [10]. The apparent diffusion coefficient (ADC) is a quantitative measure of water molecule diffusion in tissues. Low values indicate restriction (<1.0 x 10-3 mm^2^/s), varying by organ and pathology [11,12].

In treatment, MRI is useful with protocols for neuronavigation in patients undergoing surgical resection [13]. For stereotactic radiosurgery (SRS), it is recommended to perform MRI with a postcontrast T1 3D sequence 1-2 weeks before SRS, with follow-up every 2-3 months [14,15]. It is important to know if patients are receiving immunotherapy as it is associated with pseudoprogression [2]. The SRS treatment protocol considers the number, size, location, and clinical condition of the patient. A stereotactic localization system is used to precisely guide radiation to brain lesions [14,16].

For posttreatment response assessment, the RANO-BM criteria are used to establish four response categories: complete response, partial response, stable disease, and disease progression [17]. During follow-up, radiological changes in the tumor can represent progression or pseudoprogression. An increase in tumor volume can represent a mixture of progression and radiation necrosis. An initial increase in imaging abnormalities such as the enhancement pattern, followed by a subsequent decrease during follow-up in a relevant clinical period (3-6 months) should be considered as pseudoprogression [2,18,19].

The T1/T2 mismatch is a parameter that helps differentiate radiation effects from tumor progression. It corresponds to the absence of a clear and defined lesion margin on T2-weighted images compared to the contrast enhancement margin on T1-weighted images. Conversely, the T1/T2 match is associated with tumor progression and occurs when there is a correspondence between the enhancement margin of the lesion on T1 and the defined low signal margin of the lesion on T2 [20].

The response to treatment of metastatic lesions is an area under study. Due to the highly variable behavior of these lesions, more information needs to be gathered. Interpreting changes and behavior of lesions postradiosurgery can be a diagnostic challenge and can directly impact therapeutic decision-making for the patient. Proper analysis of these findings allows for better guidance on the type of treatment response exhibited by the lesions, enabling the continuation or modification of the patient's treatment course accordingly. The aim of this study was to evaluate the morphological changes and molecular behavior of BMs postradiosurgery using MRI to assess treatment response, including DWI, SWI and T1/T2 matching, which are simple and quick evaluation methods.

## Materials and methods

Patients

This is an observational, retrospective, cross-sectional survey study approved by the Institutional Research and Ethics Committee. Patient data was kept anonymous to respect the institution's privacy policy. The integrity and safety of the patients were not compromised during this study.

Access to the radiosurgery department's database was requested, obtaining 246 patients diagnosed with BMs and managed with targeted radiosurgery, covering the period from January 2018 to September 2023. A search was then conducted in the Carestream Vue PACS system to verify those patients who had complete studies performed at our institution. The MOSAIQ system was also reviewed to search for the doses used and to determine which lesions were treated. Patients who did not have baseline pre-SRS studies, follow-ups at 2-3 (MRI-2) and 5-6 months (MRI-3), as well as those with only one metastatic lesion who received surgical management as the primary therapeutic measure with a single session of radiosurgery for consolidation, were excluded. A database was created with the information and characteristics of the patients, resulting in a final sample of 41 patients. Primary tumor type, total number of intracranial lesions, systemic treatment type, and radiation parameters were recorded for each patient. The inclusion and exclusion criteria are presented in Table 1.

**Table 1:** Inclusion and exclusion criteria MRI: Magnetic resonance imaging; SRS: stereotactic radiosurgery

Inclusion criteria	Exclusion criteria
Patients with brain metastatic lesions who received SRS as the main management	Patients with a single metastatic lesion who received surgical management as the primary treatment with consolidation through radiosurgery
Patients with surgical resection of the dominant metastatic lesion with SRS treatment of the remaining lesions	Patients without follow-up studies at 2-3 and 5-6 months or with studies from another institution
MRI with a radiosurgery protocol before and after management, with follow-ups at 2-3 and 5-6 months	

Imaging acquisition and MRI protocol

All patients had high-resolution baseline pre-SRS and follow-up studies, which includes 3D sagittal T2 SPACE, axial T2, axial FLAIR, axial DWI, axial SWI, 3D axial T1 magnetization prepared-rapid gradient echo (MPRAGE) pre- and postcontrast with gadolinium. The exams were performed on a 3 Tesla Siemens MAGNETOM Skyra scanner using a 16-channel head coil. The MRI protocol is summarized in Table 2.

**Table 2:** Basic institutional MRI protocol for SRS and follow-up FLAIR: Fluid-attenuated inversion recovery; DWI: diffusion-weighted imaging; SWI: susceptibility-weighted imaging; MRI: magnetic resonance imaging; SRS: stereotactic radiosurgery; MPRAGE: magnetization prepared-rapid gradient echo

Sequence	TE (ms)	TR (ms)	TI (ms)	Flip angle	Slice thickness (mm)	Matrix
Sagital T2 SPACE	408	3200			0,90	256 x 256
Axial T2	97	5800		150°	4	512 x 384
Axial FLAIR	84	9000	2500	150°	4	320 x 217
Axial DWI	95	3800		180°	4	224 x 224
Axial SWI	20	27		15°	1.5	256 x 223
Axial 3D T1 MPRAGE	2,47	2200	900	8°	1	256 x 256
Axial 3D T1 MPRAGE +G	2,47	2200	900	8°	1	256 x 256

Radiation therapy

All lesions were treated using a Novalis Tx linear accelerator (LINAC) with a robotic table, and a 2.5 mm multileaf collimator (MLC) that allows for adjusting and shaping the radiation field to match the lesion, an ExacTrac verification system to align and verify the patient’s position before and during treatment, as well as cone-beam computed tomography (CBCT) that provides three-dimensional images for precise guidance and verification during radiotherapy treatment were used. To immobilize the patient, a frame or a specific thermoplastic mask for radiosurgery is utilized. Additionally, the Brainlab Elements Multiple Brain Mets SRS system is used to create radiosurgery plans for the simultaneous treatment of multiple lesions by performing automatic volumetric planning based on predefined protocols. It performs image fusion with presurgical contrast-enhanced MRI, segmentation of targets and organs at risk, with automatic dose distribution and margins adapted considering the location and size of the lesion. All cases were treated after being presented to the SRS committee and accepted by at least one radiation oncologist and one neurosurgeon, after which the treatment plan is reviewed. The radiation ranges used were from 14 Gy to 30 Gy with single and fractionated doses, depending on the size and location of the lesions.

Image interpretation

Studies were read in consensus by three radiologists, two fellowship trained with five years of experience, and one fellowship trained with eight years of experience, analyzing the morphological characteristics pre- and posttreatment, including if the lesion was solid, cystic, predominately solid or cystic, enhancement pattern (solid homogeneous or heterogeneous, nodular type considering a size of ≤1 cm, ring enhancement with a thickened area, cystic with mural nodule, partial solid-cystic component (50%-50%), no enhancement), number of lesions, size, volume, presence and distribution of susceptibility areas, diffusion restriction, ADC value, matching T1/T2 and response using the RANO-BM criteria (complete response, partial response, stable disease, progressive disease) for each follow-up. For lesion analysis, measurable or target lesions according to RANO-BM criteria (minimum size of 10 mm in maximum longitudinal diameter and at least 5 mm in perpendicular diameter) were selected in all patients; following this criteria, we obtained a total of 41 measurable lesions in 41 patients.

Statistical analysis

For the population description, an analysis of central tendency measures was performed using means and medians according to the data distribution, acquiring frequencies, standard deviation (SD), and interquartile ranges (IQR, 25%-75%). In the second part of the statistical analysis, logistic regression was performed relating the T1/T2 matching and the SWI distribution for MRI-2 and MRI-3. Cross tables were also made concerning treatment response and demographic characteristics according to Pearson's Chi-squared test. A p < 0.05 was used to indicate statistical significance. The statistical analysis was performed using IBM SPSS Statistics for Windows, Version 23 (Released 2015; IBM Corp., Armonk, New York, United States).

## Results

As shown in Table 3, a total of 41 patients were included, with a mean age of 56.7 ± 13 years (95% CI: 52.4-60.8). Of these, 46.3% (19/41) were males and 53.7% (22/41) were females. The most common primary tumors were lung at 29.3% (12/41), breast at 19.5% (8/41), colon at 12.2% (5/41), and melanoma and kidney tumors at 7.3% (3/41). The radiation doses used ranged from 14 Gy to 30 Gy, with single and fractionated doses applied depending on lesion size and location. A total of 78% (32/41) received a single dose, 9.8% (4/41) received fractionated doses, and 12.2% (5/41) received a single dose for some lesions and fractionated doses for larger lesions. A total of 39% (16/41) received systemic treatment with chemotherapy plus targeted therapy, 29.3% (12/41) received chemotherapy alone, 24.4% (10/41) were treated with targeted therapy, and 7.3% (3/41) received targeted therapy plus immune checkpoint inhibitors.

**Table 3:** General characteristics of the population ^1^Normally distributed variable. ^2^Freely distributed variable

Variable	Mean ± SD
Age^1^	56.7 ± 13
	95% CI (52.4-60.8)
	Frequency % (number of patients/total n = 41)
Gender^2^	Men: 46.3 (19)
	Women: 53.7 (22)
Primary tumour^2^	Lung: 29.3 (12)
	Breast: 19.5 (8)
	Colon: 12.2 (5)
	Melanoma: 7.3 (3)
	Renal: 7.3 (3)
	Esophagogastric junction: 4.9 (2)
	Endometrial: 2.4 (1)
	Esophageal: 2.4 (1)
	Bladder: 2.4 (1)
	Oropharyngeal: 2.4 (1)
	Cervix: 2.4 (1)
	Face and scalp: 2.4 (1)
	Head and neck: 2.4 (1)
	Synchronous (breast and lung): 2.4 (1)
Type of radiation dose^2^	Single dose: 78 (32)
	Fractionated dose: 9.8 (4)
	Single and fractionated dose: 12.2 (5)
Type of associated therapy^2^	Chemotherapy: 29.3 (12)
	Targeted therapy: 24.4 (10)
	Chemotherapy + targeted therapy: 39 (16)
	Targeted therapy + immune checkpoint inhibitors: 7.3 (3)

Table 4 shows the size, number, and volume of the lesions as observed on MRI. The total number of lesions in the baseline MRI prior to radiosurgery were 245, with a median of five (IQR: 1.0-8.5), the highest number of lesions in a single patient being 28. The major axis of the lesions had a mean of 19.5 ± 9.1 mm (95% CI: 16.6-22.4 mm), and the volume had a median of 2.6 cc (IQR 1.3-5.2 cc). In the second MRI, taken 2-3 months after radiosurgery, the total number of lesions were 265, the median number of total lesions was five (IQR 2.0-9.5), with the highest number being 28. The major axis had a mean of 12.8 ± 7.2 mm (95% CI: 10.5-15.1 mm), and the median volume was 0.83 cc (IQR: 0.34-2.7 cc). In the third MRI, taken 5-6 months after radiosurgery, the total number of lesions were 303, the median number of total lesions was seven (IQR 2.0-10.0), with the highest number being 25. The major axis had a mean of 11.9 ± 7.5 mm (95% CI: 9.5-14.3 mm), and the volume had a median of 0.85 cc (IQR: 0.24-2.2 cc).

**Table 4:** Number, size, and volume of lesions by MRI study MRI​​​​​​: Magnetic resonance imaging​​; SD: standard deviation; IQR: interquartile range*​​​​​* ^1^Normally distributed variable, ^2f^reely distributed variable, ^3^SD, ^4^IQR

Variable	Baseline MRI		MRI-2		MRI-3	
	Mean/median	SD^3^/IQR^4 ^(25%-75%)	Mean/median	SD/IQR(25%-75%)	Mean/median	SD/IQR(25%-75%)
Number of lesions (highest number in one patient)^2^	5 (28)	1.0-8.5	5 (28)	2.0-9.5	7 (25)	2.0-10.0
Major axis (mm)^1^	19.5	9.1 (CI 95%: 16.6-22.4)	12.8	7.2 (CI 95%: 10.5-15.1)	11.9	7.5 (CI 95%: 9.5-14.3)
Volume (per lesion, cc)^2^	2.6	1.3-5.2	0.83	0.34-2.7	0.85	0.24-2.2
	Min: 0.08		Min: 0.0		Min: 0.0	
	Max: 25		Max: 35		Max: 34	

Regarding the morphology of the lesions, it was observed that in the baseline MRI, 65.9% (27/41) were solid, 26.8% (11/41) were predominantly solid, and 7.3% (3/41) were predominantly cystic. In the second MRI, 41.5% (17/41) were predominantly cystic, 29.3% (12/41) were predominantly solid, and 24.4% (10/41) were solid; in 4.9% of the cases, the measurable lesions had disappeared or were absent. In the third MRI, 41.5% (17/41) of the lesions were predominantly cystic, 22% (9/41) were solid, 19.5% (8/41) were predominantly solid, 9.8% (4/41) were cystic, and 7.3% (3/41) were absent.

The enhancement pattern in the baseline MRI was predominantly solid heterogeneous in 43.9% (18/41) of the lesions, followed by 34.1% (14/41) homogeneous solid, 14.6% (6/41) nodular (considering a size ≤ 1 cm), 4.9% (2/41) ring enhancement with thickened area, and 2.4% (1/41) cystic with a mural nodule.

In MRI-2, 41.5% (17/41) of the lesions showed enhancement with a reduction in the solid component, tending toward a cystic pattern, 17.1% (7/41) were nodular, 17.1% (7/41) had ring enhancement with thickening, 12.2% (5/41) were heterogeneous solid, 7.3% (3/41) were homogeneous solid, and 4.9% (2/41) showed no enhancement.

In the third MRI, 26.8% (11/41) exhibited ring enhancement with thickening, 19.5% (8/41) showed a reduction in the solid component with a tendency toward a cystic pattern, 14.6% (6/41) were nodular, 14.6% (6/41) showed a partial solid and cystic component, 9.8% (4/41) had ring enhancement, 7.3% (3/41) were homogeneous solid, and 7.3% (3/41) showed no enhancement.

Table 5 presents the distribution of perilesional edema, behavior on DWI, ADC map, and SWI, as well as the type of T1/T2 matching obtained and the treatment response according to the RANO-BM criteria for each study. In the baseline MRI, perilesional edema was observed in 100% (41) of the lesions, 70.7% (29/41) showed facilitated diffusion, and 29.3% (12/41) had diffusion restriction. The ADC values had a mean of 1.06 ± 0.25 (95% CI: 0.9-1.1). SWI evaluation showed absent areas of magnetic susceptibility in 43.9% (18/41), a peripheral distribution in 26.8% (11/41), a central-peripheral distribution in 19.5% (8/41), and a central distribution in 9.8% (4/41).

**Table 5:** Morphological and molecular characteristics of lesions by MRI MRI: Magnetic resonance imaging; RANO-BM: response assessment in neuro-oncology brain metastases; DWI: diffusion-weighted imaging; SWI: susceptibility-weighted imaging ^1^Normally distributed variable. ^2^Freely distributed variable

Variable	Frequency		
	Baseline MRI	MRI-2	MRI-3
Associated edema^2^	100% (41)	No change: 2.4% (1/41)	No change: 39% (16/41)
		Decrease: 87.8% (36/41)	Decrease: 31.7% (13/41)
		Increase: 9.8% (4/41)	Increase: 29.3% (12/41)
DWI^2^			
Facilitated diffusion	70.7% (29/41)	80.5% (33/41)	78.0% (32/41)
Diffusion restriction	29.3% (12/41)	19.5% (8/41)	22.0% (9/41)
ADC^1^	Mean ± SD: 1.06 ± 0.25 (95% CI: 0.9-1.1)	Mean ± SD: 1.1 ± 0.46 (95% CI: 0.9-1.2)	Mean ± SD: 1.1 ± 0.48 (95% CI: 0.9-1.2)
SWI (distribution)^2^			
Absent	43.9% (18/41)	19.5% (8/41)	22.0% (9/41)
Central	9.8% (4/41)	4.9% (2/41)	4.9% (2/41)
Peripheral	26.8% (11/41)	31.7% (13/41)	22.0% (9/41)
Central and peripheral	19.5% (8/41)	43.9% (18/41)	51.2% (21/41)
SWI change^2^			
No change		24.4% (10/41)	65.9% (27/41)
Increase		70.7% (29/41)	26.8% (11/41)
Decrease		4.9% (2/41)	7.3% (3/41)
T1/T2 matching^2^			
Absent (T1/T2 match)		14.6% (6/41)	7.3% (3/41)
Mixed (T1/T2 mismatch + match)		70.7% (29/41)	68.3% (28/41)
Complete (T1/T2 mismatch)		9.8% (4/41)	17.1% (7/41)
No lesion		4.9% (2/41)	7.3% (3/41)
Progression^2^			
Absent		73.2% (30/41)	56.1% (23/41)
Increase in number of lesions		26.8% (11/41)	24.4% (10/41)
Increase in lesion size		0.0%	9.8% (4/41)
Increase in number and size		0.0%	9.8% (4/41)
RANO-BM^2^			
Complete response		4.9% (2/41)	2.4% (1/41)
Partial response		43.9% (18/41)	34.1% (14/41)
Stable disease		22.0% (9/41)	19.5% (8/41)
Disease progression		26.8% (11/41)	43.9% (18/41)
Pseudoprogression		2.4% (1/41)	0.0%

In the second MRI, perilesional edema decreased in 87.8% (36/41) of cases and increased in 9.8% (4/41). Facilitated diffusion was seen in 80.5% (33/41) of the lesions, 19.5% (8/41) presented molecular restriction, and the mean ADC value was 1.1 ± 0.46 (95% CI: 0.9-1.2). On SWI, areas of magnetic susceptibility with central and peripheral distribution were observed in 43.9% (18/41), peripheral distribution in 31.7% (13/41), absent in 19.5% (8/41), and central in 4.9% (2/41). Overall, there was a 70.7% (29/41) increase in areas of susceptibility and a 4.9% decrease (2/41). The T1/T2 matching pattern showed a mixed pattern (areas of match and mismatch T1/T2) in 70.7% (29/41) of the lesions, 14.6% (6/41) had T1/T2 match, 9.8% (4/41) had complete T1/T2 mismatch, and in 4.9% (2/41), it was not measurable due to lesion disappearance. Regarding treatment response based on the RANO-BM criteria, 43.9% (18/41) had a partial response, 22% (9/41) had stable disease, 4.9% (2/41) had a complete response, and 26.8% (11/41) showed disease progression, mostly due to an increase in the number of lesions. In 2.4% (1/41), pseudoprogression was observed due to the characteristics of the lesions and the type of systemic treatment (targeted therapy + immune checkpoint inhibitors).

In the third MRI, 39% (16/41) of the lesions showed no change in perilesional edema, 31.7% (13/41) had a reduction in edema, and 29.3% (12/41) showed an increase. Facilitated diffusion was observed in 78% (32/41) of the lesions, while 22% (9/41) had diffusion restriction. The ADC value had a mean of 1.1 ± 0.48 (95% CI: 0.9-1.2). On SWI, areas of magnetic susceptibility with central and peripheral distribution were seen in 51.2% (21/41), peripheral distribution in 22% (9/41), and absence of susceptibility in 22% (9/41), with 4.9% (2/41) showing a central pattern. Overall, there was a 26.8% (11/41) increase in areas of magnetic susceptibility, a 7.3% (3/41) decrease, and 65.9% (27/41) showed no change. The T1/T2 matching was mixed in 68.3% (28/41) of the lesions, complete in 17.1% (7/41), T1/T2 match was observed in 7.3% (3/41), and it was not measurable due to the absence of lesions in 7.3% (3/41). The treatment response according to the RANO-BM criteria showed disease progression in 43.9% (18/41) of the cases (mostly due to an increase in the number of lesions), 34.1% (14/41) had a partial response, 19.5% (8/41) had stable disease, and 2.4% (1/41) had a complete response.

Subsequently, a logistic regression was performed, relating the T1/T2 matching and the SWI, taking into account the major axis of the lesion for both MRI-2 and MRI-3, to assess differences based on the SWI distribution findings in relation to T1/T2 match, mixed T1/T2 matching, and complete T1/T2 mismatch, considering the total number of lesions, showing how far the mean extended.

**Figure 1:**
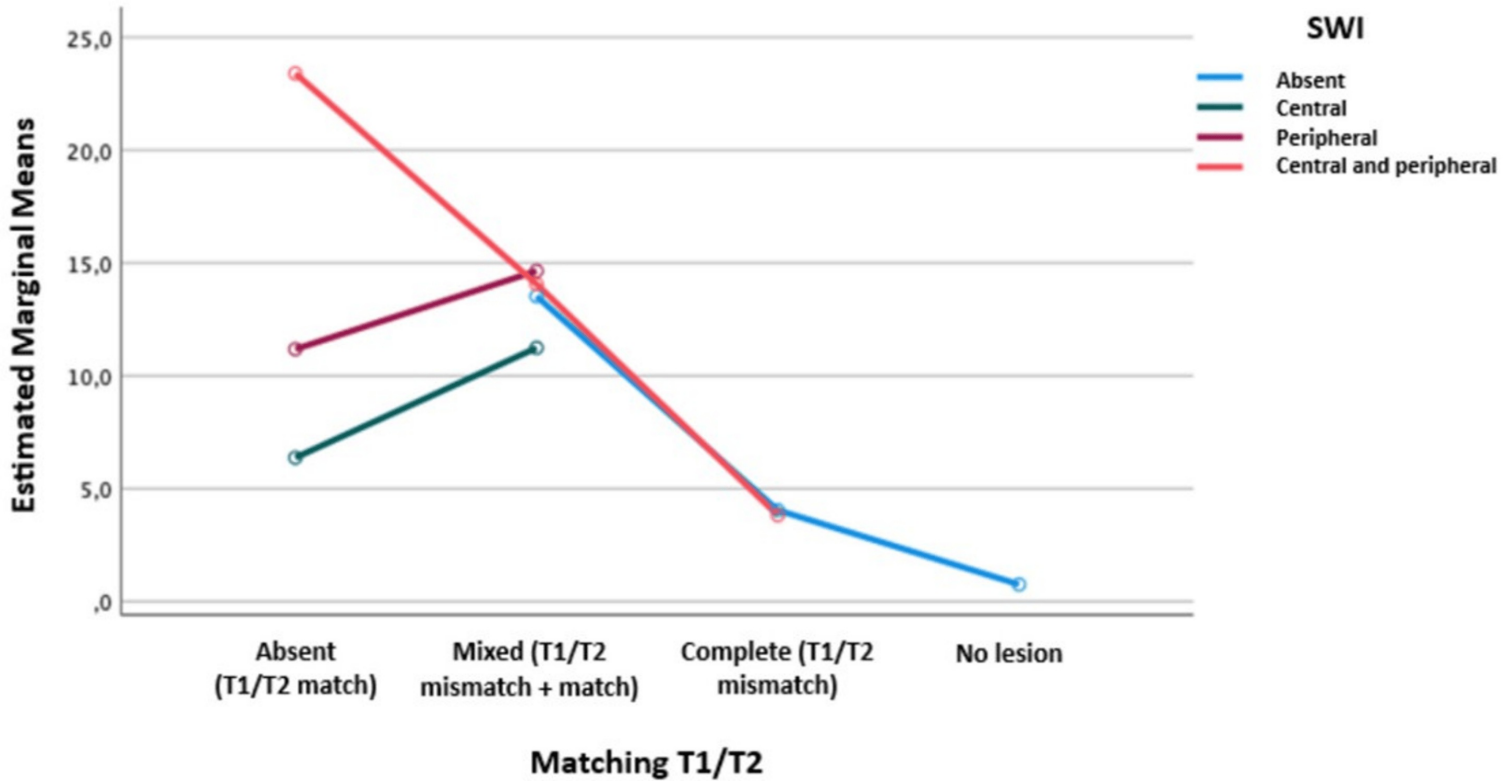
Graph shows logistic regression test relating T1/T2 matching and SWI in MRI-2 SWI: Susceptibility-weighted imaging; MRI: magnetic resonance imaging Statistical test: Logistic regression, p-value of 0.005, R² of 0.52, adjusted for the total number of lesions, relating T1/T2 matching and SWI, taking into account the major axis of the lesion

In Figure 1, MRI-2 is represented, where it was identified that when there were areas of magnetic susceptibility with central and peripheral distribution on SWI, there was a higher mean when there was a T1/T2 match. Similarly, the other SWI distributions showed a higher mean for the mixed T1/T2 matching. This model explains 52% of the findings, indicating that more than half of the time, there will be a relationship between the SWI and the T1/T2 matching, considering the total number of lesions.

**Figure 2:**
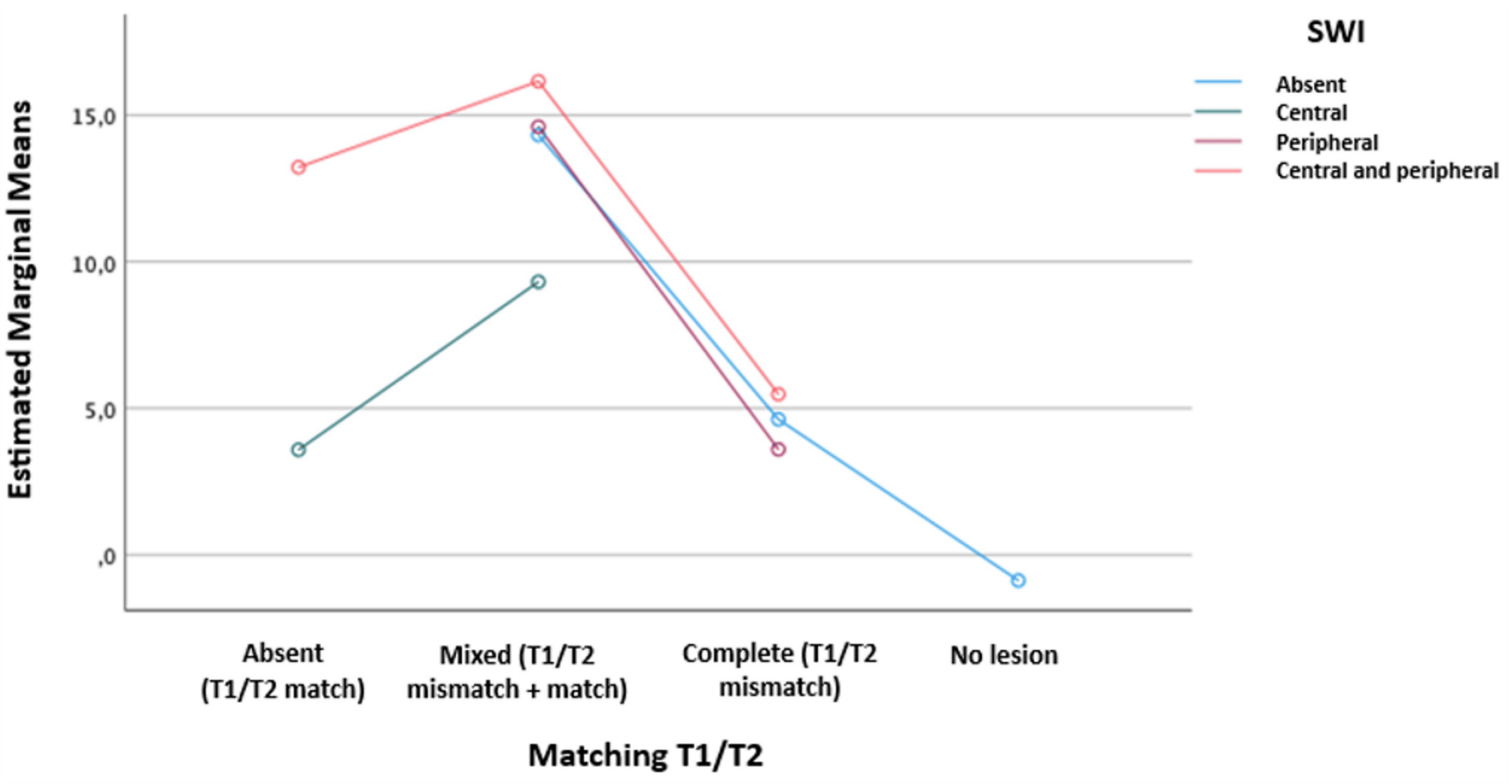
Graph shows logistic regression test relating T1/T2 matching and SWI in MRI-3 SWI: Susceptibility-weighted imaging; MRI: magnetic resonance imaging Statistical test: Logistic regression, p-value of 0.002, R² of 0.56, adjusted for the total number of lesions, relating T1/T2 matching and SWI, taking into account the major axis of the lesion

In Figure 2, for MRI-3, it is shown that when there are areas of magnetic susceptibility with central and peripheral distribution, there are higher means when there’s T1/T2 match or mixed matching. Here, as well, the other SWI distributions showed a higher mean for the mixed T1/T2 matching. When mismatch was present, SWI in almost all its distributions showed a lower mean. In this same graph, this model explains 56% of the findings.

Cross tables were created based on Pearson’s Chi-squared test, relating treatment response according to the RANO-BM criteria with the primary tumor, the systemic therapy administered, and the type of dose used in SRS for MRI-2 and MRI-3.

In MRI-2, the relationship between the primary tumor and treatment response resulted in a Chi-squared value of 0.481. Complete response was observed in lung cancer (2.4%, 1/41) and renal cancer (2.4%, 1/41). Partial response was mainly observed in breast (12.2%, 5/41) and lung cancer (14.6%, 6/41). For stable disease, lung cancer (9.8%, 4/41) was the most frequent, followed by melanoma (4.9%, 2/41). For disease progression, breast, renal, and lung cancers were the most frequent with the same percentage (4.9%, 2/41). Regarding systemic treatment and response type, a significant Chi-squared value of <0.001 was obtained. For partial response, most patients received chemotherapy + targeted therapy (29.3%, 12/41), followed by chemotherapy (9.8%, 4/41) and targeted therapy (4.9%, 2/41). Among those with disease progression, the majority received chemotherapy in 12.2% (5/41) and targeted therapy in 12.2% (5/41). For stable disease, 7.3% (3/41) received chemotherapy, 7.3% (3/41) targeted therapy, or a combination of both 7.3% (3/41). For complete response, 2.4% (1/41) received targeted therapy and 2.4% (1/41) chemotherapy + targeted therapy.

In MRI-3, the relationship between the primary tumor and treatment response showed a Chi-squared value of 0.222. Disease progression was most frequent in breast cancer (12.2%, 5/41) and lung cancer (7.3%, 3/41). Partial response was observed in lung (12.2%, 5/41), breast (7.3%, 3/41), and colon (7.3%, 3/41) cancers. For stable disease, lung cancer (9.8%, 4/41) and melanoma (4.9%, 2/41) were the most frequent, while complete response was only observed in renal tumor (2.4%, 1/41). The relationship between systemic treatment and treatment response resulted in a Chi-squared value of 0.295. For disease progression, 17.1% (7/41) received chemotherapy + targeted therapy, followed by chemotherapy and targeted therapy in 12.2% (5/41). For partial response, the majority received chemotherapy + targeted therapy (19.5%, 8/41) and chemotherapy (12.2%, 5/41). For stable disease, 7.3% (3/41) received targeted therapy and 4.9% (2/41) received chemotherapy. For complete response, 2.4% (1/41) received targeted therapy.

**Figure 3:**
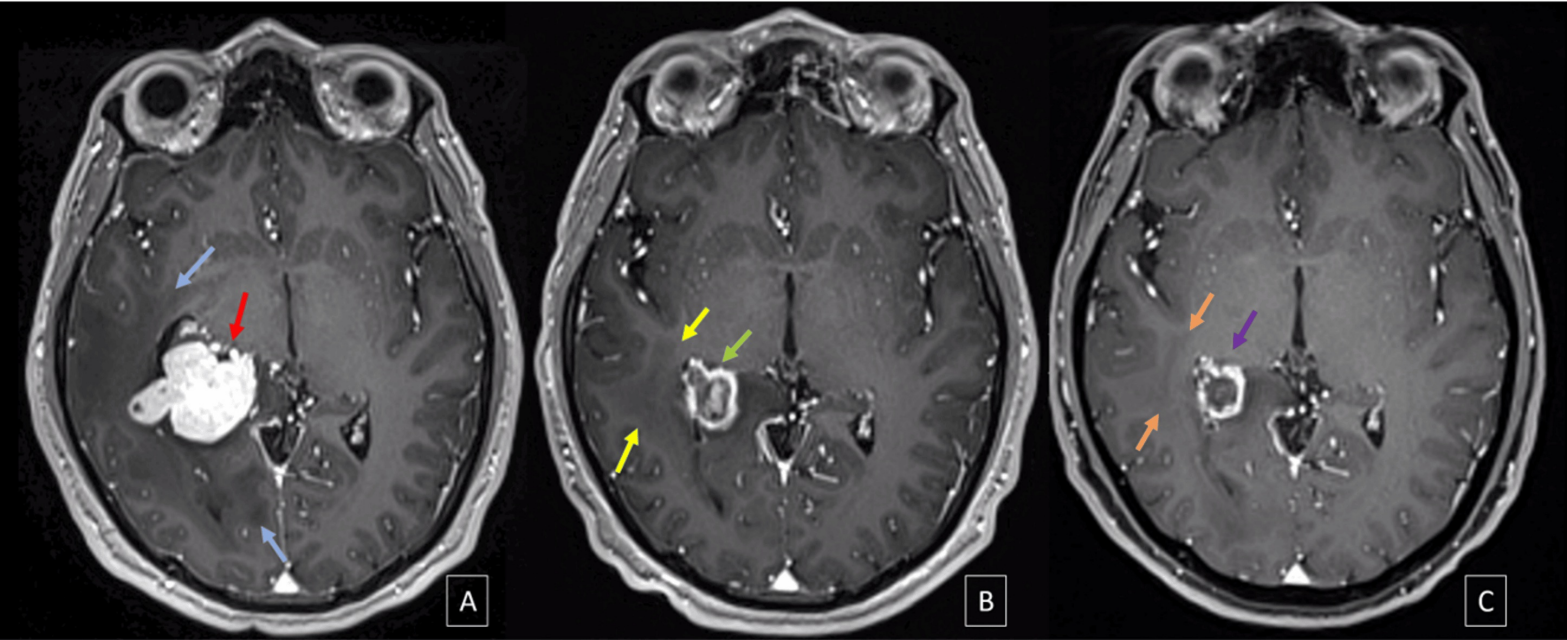
3D axial T1 MPRAGE G+ of a 41-year-old male patient with brain metastasis from clear cell renal carcinoma MPRAGE: Magnetization prepared-rapid gradient echo; MRI: magnetic resonance imaging; SRS: stereotactic radiosurgery In the baseline MRI (A), prior to SRS, a predominantly solid lesion with a homogeneous enhancement pattern is observed (red arrow), along with associated perilesional edema (blue arrows). In MRI-2 (B) at three months, there is a reduction in lesion size, with a decrease in the solid component, trending toward a cystic form due to progressive intralesional cavitation and the development of central necrosis (green arrow), as well as a decrease in perilesional edema (yellow arrows). In MRI-3 (C) at six months, further reduction in perilesional edema is seen (orange arrows), along with a decrease in lesion size, with a ring enhancement pattern, areas of peripheral thickening, and an increased central necrotic component (purple arrow)

**Figure 4:**
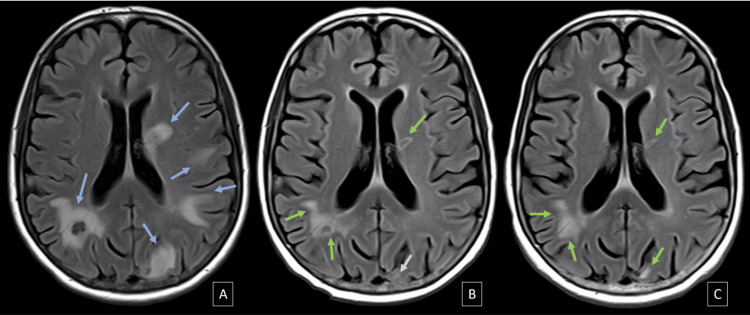
Axial FLAIR of a 64-year-old female patient with metastatic lesions from undifferentiated large cell lung cancer FLAIR: Fluid-attenuated inversion recovery; MRI: magnetic resonance imaging; SRS: stereotactic radiosurgery In the baseline MRI (A), prior to SRS, multiple areas of pronounced perilesional edema surrounding the metastatic lesions are shown (blue arrows). In MRI-2 (B) at three months and MRI-3 (C) at six months, there is a progressive decrease in the distribution and extent of perilesional edema, associated with a reduction in lesion size (green arrows)

**Figure 5:**
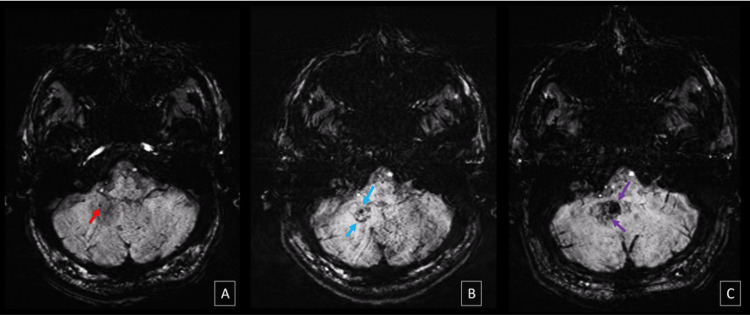
Axial SWI of a 63-year-old male patient with brain metastasis from a neuroendocrine tumor of the esophagogastric junction SWI: Susceptibility-weighted imaging; MRI: magnetic resonance imaging; SRS: stereotactic radiosurgery In the baseline MRI (A), prior to SRS, a solid lesion with punctate central magnetic susceptibility areas is observed (red arrow). In MRI-2 (B) at three months, there is a reduction in lesion size with an increase in magnetic susceptibility areas, showing central and peripheral distribution (blue arrows). In MRI-3 (C) at six months, a greater central and peripheral component of magnetic susceptibility areas is observed (purple arrows)

The type of dose used in SRS in relation to treatment response for MRI-2 (Chi-squared value of 0.620) and MRI-3 (Chi-squared value of 0.978) was predominantly single-dose. Figures 3-5 illustrate the main morphological changes, perilesional edema distribution, and areas of magnetic susceptibility following radiosurgery treatment.

**Figure 6:**
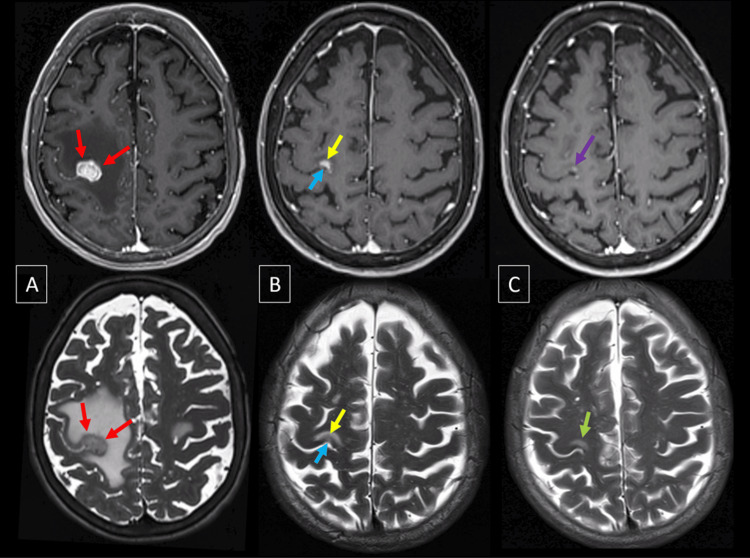
Axial 3D T1 MPRAGE G+ and axial T2 of a 67-year-old female patient with brain metastasis from non-small cell lung cancer MPRAGE: Magnetization prepared-rapid gradient echo; MRI: magnetic resonance imaging; SRS: stereotactic radiosurgery In the baseline MRI (A), a solid lesion is observed, showing a correlation between its postcontrast enhancement margin in T1 and the margin in T2 (T1/T2 match) (red arrows), along with pronounced perilesional edema. In MRI-2 (B) at three months, there is a decrease in size, with areas showing an indistinct margin in postcontrast T1 and T2 (T1/T2 mismatch) (blue arrows), along with other areas of T1/T2 match (yellow arrows). In MRI-3 (C) at six months, the lesion and its margins are not well-defined in T2 in relation to the nodular enhancement area in T1 (purple arrow), showing a complete T1/T2 mismatch (green arrow)

Figure 6 illustrates the T1/T2 matching behavior following radiosurgery treatment.

## Discussion

In this study, we observed that SWI and T1/T2 mismatch are valuable tools for detecting tumor microenvironment changes, such as microhemorrhage and radionecrosis after radiosurgery. We also found that the appearance of susceptibility areas may precede changes in the enhancement of the lesion margin post-SRS.

Different studies have been conducted to specify the epidemiological characteristics of metastatic cancer, one of the largest being the study conducted by the Metropolitan Detroit Cancer Surveillance System, which examined primary tumors and their metastases in more than 16,000 patients, finding that women, individuals aged 40-49 with lung cancer, 50-59 with melanoma, and 20-39 with breast cancer had the highest incidence of brain metastases [5,21]. In our study, the mean patient age was 56.7 years, with more than half being women. The most common primary tumors were lung, breast, colon, melanoma, and kidney tumors, similar to what has been reported in the literature.

The biological effects of radiosurgery include an early stage with edema in the central region of the treated area, an intermediate stage with progressive cavitation and capillary proliferation surrounding a necrotic center, accompanied by ring enhancement and mild perilesional edema, and a late stage with central necrosis and a glial scar with peripheral enhancement without perilesional edema. Post-SRS, between two and six months, lesions show perilesional edema, ring enhancement, and central hypointensity on T2. edema decreases over time due to the formation of a glial scar and a reduction in tumor volume [22,23]. In our study, most lesions were solid in the baseline MRI, with a heterogeneous or homogeneous solid enhancement pattern and perilesional edema. After SRS, the lesions underwent a morphological change, becoming predominantly cystic, with a decrease in perilesional edema in most cases in MRI-2, and either no change or further reduction in MRI-3. Both the major axis and volume of the lesions tended to decrease. These changes follow the pattern reported in the literature regarding the biological effects of radiation, with the development of central cavitation, necrotic core formation, and a reduction in perilesional edema and tumor size.

Kaur et al. describe that changes in peritumoral edema can be predicted within the first three months post-SRS. Lesions with peritumoral edema regression in the first follow-up (0-90 days) mostly continue to decrease. They mention that early tumor size reduction, along with decreased edema within the first three months, is associated with local control [24,25].

Most of the lesions in our study showed facilitated diffusion with ADC values above one in the baseline MRI and throughout the follow-up. However, these changes were not decisive for lesion assessment in our work. The literature suggests that ADC values can be useful for differentiating between tumor recurrence and radionecrosis after SRS, with established ADC cutoff values of 900-1000 x 10-6 mm^2^/s for distinguishing true progression from pseudoprogression [22,26]. Since measurements depend on the region of interest and its reproducibility, volumetric assessment of the entire lesion would be necessary for a more accurate ADC value, which would increase the complexity of the evaluation. Chen et al. reported in their study that the development of a diffusion index, combining ADC and tumor volume at baseline, one month, and six months post-SRS, may serve as a biomarker for predicting treatment response [12].

Magnetic susceptibility areas indicate tumor microenvironment modification, representing regions of microhemorrhage or radiation-related tissue damage [8]. These findings are observed on SWI during early lesion follow-up [9]. In our study, there was a general increase in susceptibility areas in the first post-SRS follow-up. By MRI-3, no further changes were observed in more than half of the patients, and there was a slight increase in susceptibility areas, consistent with changes reported in the literature.

Kano et al. developed a visual assessment method for lesion contour, finding that a well-defined T2 margin correlating with a distinguished postcontrast T1 margin (T1/T2 match) had a high association with tumor tissue (p < .0001). In contrast, an indistinct T2 margin without correlation in postcontrast T1 (T1/T2 mismatch) was significantly associated with necrosis detection (p < .0001), showing 83% sensitivity and 91% specificity for radionecrosis detection with T1/T2 mismatch [20]. In our study, a mixed pattern (areas of T1/T2 match and mismatch) predominated in the lesion margins in both MRI-2 and MRI-3, explaining the expected post-SRS changes with necrosis development and loss of definition in the marginal enhancement areas, consistent with the morphological changes described previously.

In this study, partial response to treatment was predominant in MRI-2, while disease progression, mostly due to an increase in the number of lesions, was more common in MRI-3. Therefore, it is crucial to conduct follow-up at intervals of 2-3 months, as around half of the patients treated with SRS develop new metastases in other brain regions during the follow-up period [2].

In the logistic regression analysis relating T1/T2 matching and SWI distribution, a higher mean presentation of central-peripheral SWI distribution was observed for T1/T2 match during both follow-up periods after SRS, with a significant p-value. This may suggest that the appearance of susceptibility areas can precede changes in lesion margin enhancement. Additionally, a higher mean was observed for all SWI distribution scenarios when a mixed T1/T2 mismatch was present, indicating necrotic changes in the lesion with areas of microhemorrhage and tumor tissue damage seen as magnetic susceptibility areas [20,22].

The literature reports that the most common tumor types developing brain metastases respond better with a combination of SRS and targeted therapies, including non-small cell lung cancer, HER-2-positive breast cancer, endocrine receptor-positive breast cancer, melanoma, and renal cell carcinoma [27]. Additionally, radioresistant tumors such as melanoma and renal cell carcinoma show promising responses to SRS, with reports suggesting control rates similar to those of radiosensitive tumors (breast and lung cancer), indicating that SRS efficacy may be independent of the primary tumor [28,29]. In our study, no predominant cancer type was found for treatment response. However, our sample was small, with limited tumor variety. Only a significant p-value of <0.001 was found for systemic treatment and response type in MRI-2, with partial response predominantly in patients who received chemotherapy + targeted therapy alongside SRS. However, this combination was the most frequently used treatment among all patients, and no significant value was observed in MRI-3, possibly due to sample heterogeneity. The dose type is dependent on lesion size and location, typically involving single doses of 18-24 Gy, moderate fractionated doses of 24-27 Gy, or up to 30 Gy in five fractions [30]. In our case, no correlation was found between treatment response and the type of dose used.

Based on the findings, it is recommended that part of the follow-up protocol for patients with metastatic lesions treated with radiosurgery should include volumetric T1-weighted pre- and postcontrast sequences with gadolinium, 3D isotropic T2 SPACE or equivalent sequences, and high-resolution SWI with thin slice thickness. These are essential for accurate T1/T2 mismatch assessment and evaluation of magnetic susceptibility area distribution. A limitation of this study was the small population size; hence, a larger study is needed to further validate our results.

## Conclusions

SWI and T1/T2 mismatch are useful tools that reveal changes explaining the modification of the tumor microenvironment, associated with microhemorrhage and the development of radionecrosis following radiosurgery. The appearance of susceptibility areas may precede changes in the enhancement of the lesion margin. These changes correlate with the morphological evolution of the lesions, which tend to reduce their solid component and become cystic. These evaluation techniques are valuable for monitoring the patient's treatment response. Early reductions in lesion size and perilesional edema can help predict local control. It is essential that follow-ups after SRS be conducted at short intervals of 2-3 months, as progression is primarily due to the appearance of new lesions in approximately 50% of patients.
